# Degradation mechanisms in gate-all-around silicon Nanowire field effect transistor under electrostatic discharge stress – a modeling approach

**DOI:** 10.1186/s40580-014-0011-9

**Published:** 2014-04-24

**Authors:** Cher Ming Tan, Xiangchen Chen

**Affiliations:** School of Electrical & Electronic Engineering, Nanyang Technological University, Nanyang, 639798 Singapore

**Keywords:** ESD, Hot carrier injection, Oxide breakdown, Silicon melting, Sentaurus simulation

## Abstract

The failure and degradation mechanisms of gate-all-around silicon nanowire FET subjected to electrostatic discharge (ESD) are investigated through device modeling. Transmission line pulse stress test is simulated and device degradation physics is modeled. The device degradation level, interface state concentration and hard breakdown are shown and analyzed. From the model, we found that ESD stress can induce severe performance degradation or even hard breakdown of gate-all-around nanowire device, and the interface traps due to hot carrier injection is responsible for the device degradation.

## Background

Gate-all-around silicon nanowire (GAA SiNW) FET is a promising candidate for future scaled silicon based devices. Recent research on device fabrication and characterization demonstrate that GAA SiNW FET possesses enhanced electrical performances and good immunity against short channel effect [1]. However the gate-all-around structure with poor thermal conductivity of the gate stack material tends to confine the heat dissipated from the device itself, renders high device temperature and impact its performances and reliability. The scaled feature size makes the device to operate in high field condition and the gate wrap configuration increases the ratio of channel-dielectric interface area to nanowire channel volume. This renders the device susceptible to Si-SiO_2_ interface related degradation mechanisms, and in particular vulnerable to electrostatic discharge (ESD) stress condition. Recent experimental works demonstrate its limited ESD reliability. The ESD damaged FET degrades significantly and some of the nanowires burnout and melt due to the dramatic increased local hotspot temperature [2].

ESD degradation and breakdown mechanisms for GAA nanowire devices are expected to be different from the mechanism of standard MOSFETs. Previous ESD failure analysis study on MOS devices shows that the major failure mechanism is the second breakdown of PN junction of the transistor. For a gate grounded MOS transistor during ESD event, the drain-base PN junction is reverse biased until avalanche breakdown occurs in depletion region. The generated carriers then forward bias the source-base junction, and turns on the parasitic bipolar junction transistor to dissipate the ESD charge. The device enters into second breakdown if it fails to sustain the discharging current due to overheating [3].

In the case of GAA nanowire FET, its substrate is floating and this renders the absence of the parasitic bipolar junction transistor dissipation path in the device, and thus no snapback behavior is expected during the nanowire device breakdown. This is confirmed experimentally by [2]. However, the degradation physics in GAA FETs under ESD stress remains unexplored.

In this work, we employ TCAD Sentaurus to understand the degradation physics of GAA SiNW FET due to ESD. A model will be developed and first verified using the reported ESD experimental data in [2]. With this model, we study the degradation process in GAA FETs in order to deepen our understanding of the degradation physics in GAA SiNW FETs under ESD for defining the requirement of ESD protection circuits for GAA SiNW FET circuits in future. Only human body model (HBM) is considered in this work as the current available ESD experimental data on GAA SiNW FET is limited to HBM

A device description in Section 2 introduces the details of the device model. The simulation experiment on ESD test and degradation characterization are discussed in Section 3, and the device degradation mechanism analysis based on the simulation results are presented in Section 4.

## Methods

### Device description

The GAA SiNW FET is a SOI (silicon-on-insulator) based device structure. It uses a floating silicon nanowire as conducting channel between source and drain, and the gate oxide is wrapping around the nanowire body. In this work, the GAA SiNW FET device geometry is generated using Sentaurus Process with MGOALS3D library in this work.

**Figure 1:**
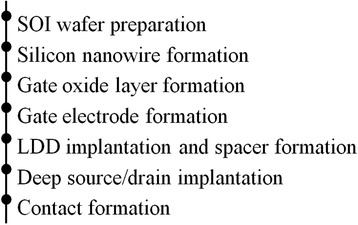
**Process steps of defining the GAA SiNW device.**

The process simulation steps are given in Figure 1 which are similar to the actual device fabrication process discussed in [1]. The gate length of the device is 50 nm, the diameter of the nanowire is 10 nm and the gate dielectric layer thickness is 3 nm. The device doping is generated by applying arsenic ion dose of 2 × 10^13^cm^-2^ for the LDD implantation and 6 × 10^15^ cm^-2^ for the source and drain implantation. The gate contact work function is set as 4.6 eV. All these parameters followed the work of [1]. To improve the simulation efficiency and numerical robustness, the device geometry is boundary conforming meshed based on active doping concentration [4].

Device simulation and characterization are performed with Sentaurus Device. The electrical behavior of nanowire device is modeled using the drift-diffusion carrier transport model with density gradient quantization correction and thermal coupling effect [5]. The thermal boundary condition is 300 K as ambient temperature, and the device thermal dissipation is via the surface thermal resistance of 5 × 10^-5^ cm^2^KW^-1^ at the drain contact [6].

On examining the device structure of GAA SiNW FETs, when a high voltage and high current are exerted on the devices during ESD, three possible degradation/damage mechanisms are possible, namely the oxide breakdown, interface traps generation and SiNW damage including the PN junction failure.

To account for all these mechanisms, the following models in Sentaurus simulation suite [4] are activated, namely the SRH model; high field mobility saturation model; the Conwell-Weissopf carrier-carrier scattering model to model the mobility degradation under high ESD current injection; the University of Bologna impact ionization model to model the impact ionization generation process at high temperature condition; thermodynamic model with absolute thermoelectric power consideration to model the heat generation and lattice hotspot movement in the device structure. The effect of Si-SiO_2_ interface trap generation based on the Si-H defect kinetics is also tracked with multiple trap generation enhancement schemes included in the model [7].

SRH and the impact ionization models have been used successfully in explaining the experimental data of Si nanowire based SONOS memory cells with nanowire diameter of 5 nm [8], and thus their applicability to nanowire modeling is verified. The high field mobility saturation model has also been used to study the electrical transport in Si nanowire [5].

Hwang et al. [9] and Novoselov et al. [10] showed that when the carrier concentration is above 10^12^ cm^-2^, full Boltzmann transport theory is still applicable for 2D electron transport, and as our nanowire doping concentration is above 10^12^ cm^-2^, especially under high ESD current injection, the use of Conwell-Weissopf carrier-carrier scattering model in our work is justified as it was derived based on the Boltzmann transport theory. The self-heating effect in this work is studied from the modeling of heat transfer based on the thermodynamics model, followed the work of Pop who studied the thermal transport in nanoscale devices, including silicon nanowire of diameter down to 22 nm [11]. The silicon nanowire thermal conductivity is also used instead of that of the bulk silicon, and its value is obtained from [11].

Therefore, all the models in Sentaurus used in this work are found applicable for the silicon nanowire studied here. Furthermore, all the above-mentioned models do not require temperature calibration except the impact ionization model. The impact ionization model used here has been calibrated from 25 – 500°C. Our worst case degradation is slightly above 500°C as will be seen later, and hence the simulation results obtained in this work should be adequately valid.

### TLP ESD simulation

**Figure 2:**
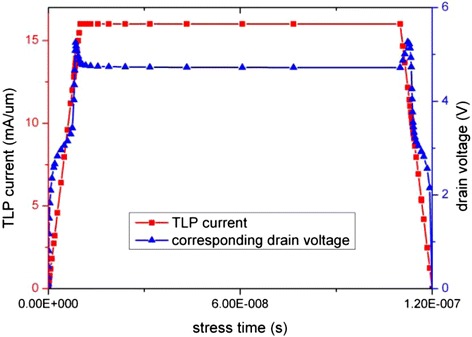
**TLP current curve and resulting drain voltage curve.**

As the current available experimental data on the ESD damage of GAA SiNW FETs were done using TLP [2,12], ESD simulation is done by applying a transmission line pulsing (TLP) input to stress the device so that model verification can be made. An n-type GAA nanowire FET is studied in this work. Following the work by [2,12], the input pulse is set as human body model (HBM) equivalent with 10 ns rising/falling time and 100 ns pulse width as shown in Figure 2. The gate contact is left floating during the TLP test [2,12].

**Figure 3:**
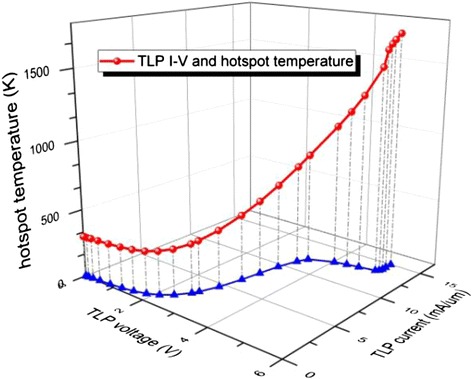
**Hotspot temperature tracking during TLP stress.**

Various pulse current levels are first applied to obtain the critical stress level that causes the device hard breakdown, i.e. the condition at which the maximum temperature in the device structure exceeds the melting point of silicon. Figure 3 shows the TLP I-V curve and the maximum device temperature (hotspot temperature) in the z-axis during the TLP. From Figure 3, we can see that when the current level is 15 mA/μm, where the normalization factor is the diameter of the nanowire, the hotspot temperature can reach as high as 1700 K during the TLP stress period, causing device hard breakdown as the temperature exceeds the melting point of silicon. This stress level, denoted as I_t2_, is the maximum current stress that can be applied which lead to catastrophic failure. The experimental stress level that causes silicon nanowire melting is 10-30 mA/μm [2,13], and we can see the close agreement of our simulation results with the experiments.

The maximum oxide electric field under the I_t2_ stress level is around 0.75 V/nm, and since gate oxide breakdown field is 1 V/nm [13], oxide breakdown is unlikely to be the dominant degradation mechanism for GAA SiNW FETs under ESD.

No physical damage but severe performance degradation is observed experimentally when the current stress is set to be two-third of I_t2_ [2]. Our simulation with the same set-up shows that the maximum silicon temperature is only 600°C which is far from silicon melting temperature, which explain the absence of physical damage. Therefore, from the above analysis, it is clear that when the current stress level is below I_t2_, the degradation mechanism is due to the interface traps generation.

Different stress levels ranging from one-sixth to two-thirds of I_t2_ are applied to study the device degradation physics. The post-degradation device characterization is conducted and compared to its characterization before the stresses.

**Figure 4:**
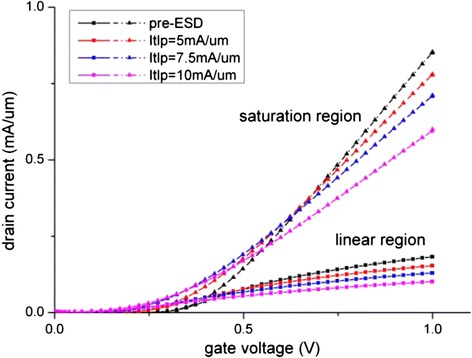
**I**_**d**_ **-V**_**g**_ **comparison between fresh and degraded devices.**

**Figure 5:**
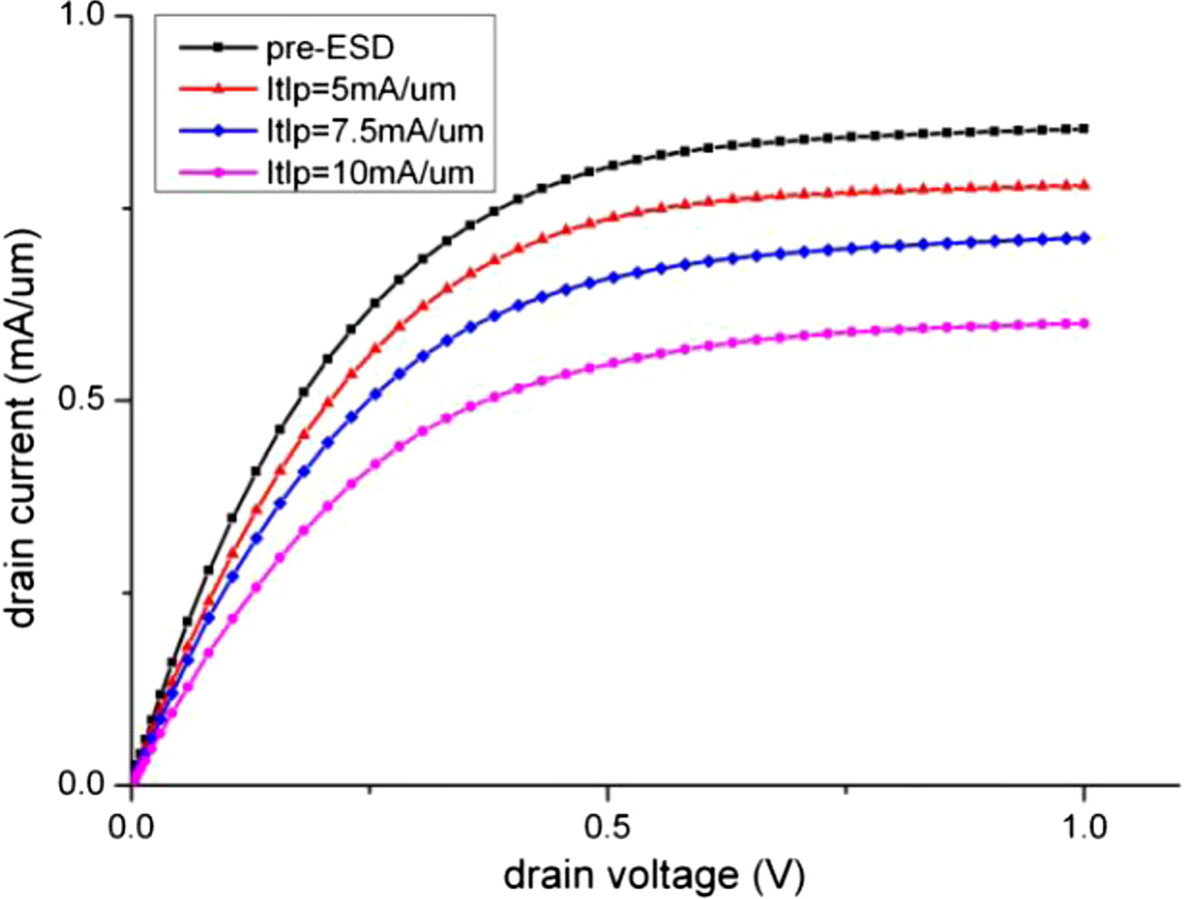
**I**_**d**_ **-V**_**d**_ **comparison between fresh and degraded devices.**

The comparison of I_d_-V_g_ and I_d_-V_d_ of pre- and post-ESD devices are depicted in Figures 4 and 5. Figure 5 shows that when the current stress is at two-third of I_t2_, i.e. 10 mA/μm, a 32% on-state current degradation is observed, and this is in good agreement of the reported experimental data of 39% [2]. This further verified that our model is sufficiently accurate and that the generation of interface traps is the dominant degradation mechanism.

Interface trap generation at Si/SiO_2_ interface due to ESD TLP has been studied by Tseng and Hwu [14–16]. They found that the bond breaking by energetic electrons through the oxide is the dominant mechanism. However, in their studies, the TLP pulse was applied at the gate and thus current did flow through the oxide and generate interface traps. In our case, our gate is floating, and the TLP pulse is applied to the drain with the source grounded. This is done to mimic the set up of the reported experimental work [2]. As there is no current flow through the oxide, the bond breaking mechanism in oxide as reported earlier by Tseng et al. [14–16] will not be possible, and the only possible source of interface trap generation in our case will be via hot carrier injection.

## Results and discussion

**Figure 6:**
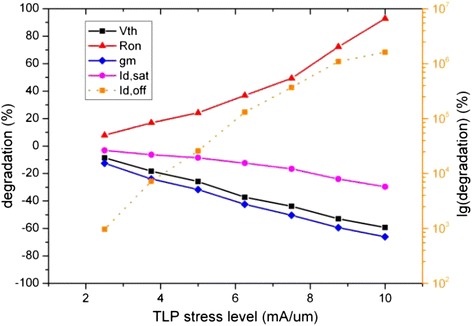
**Degradation of the device electrical parameters (the scale for the off-state drain leakage current is on the right hand side).**

**Figure 7:**
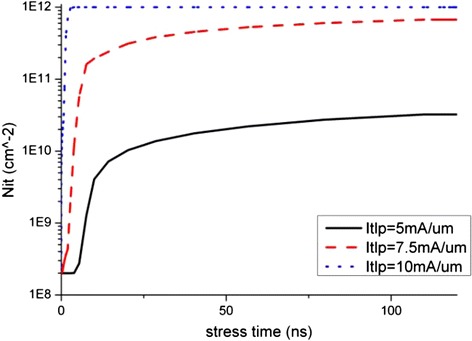
**Maximum interface trap concentration over entire interface region.**

**Figure 8:**
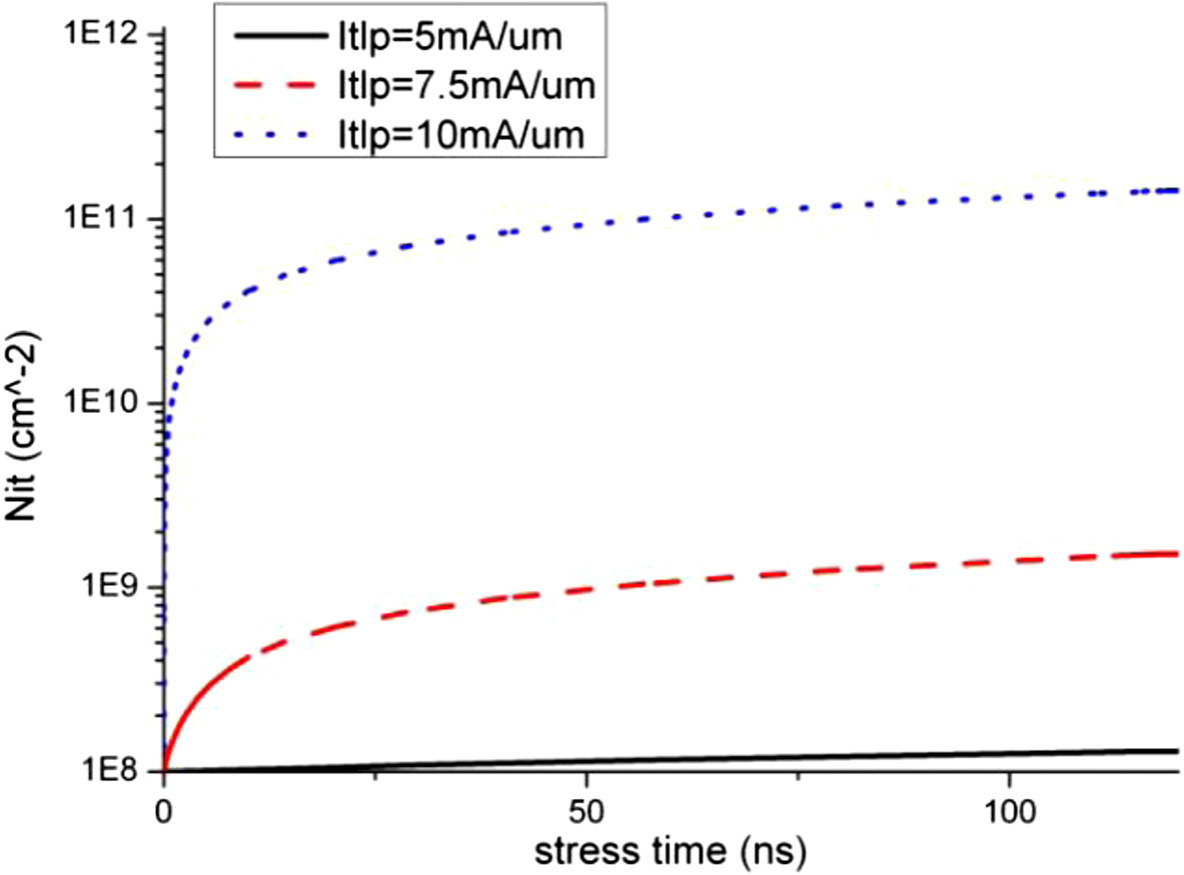
**Average interface trap concentration at the source region.**

**Figure 9:**
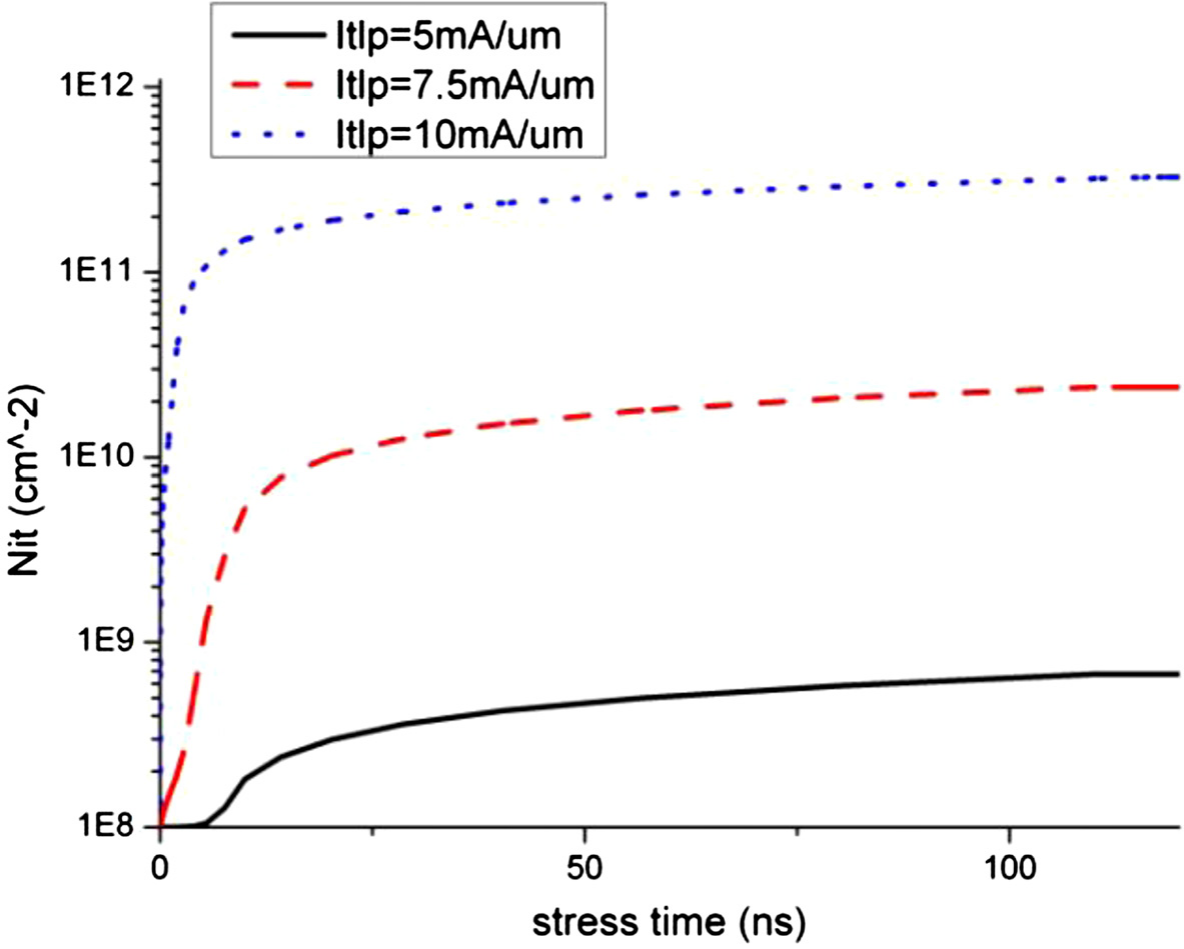
**Average interface trap concentration at the drain region.**

The degradation data of the key device electrical parameters extracted from Figures 4 and 5 are shown in Figure 6, and the time progression of interface trap generation during the TLP are shown as interface trap concentration (N_it_) versus pulse time in Figures 7, 8 and 9. The maximum trap concentration over the entire Si-SiO_2_ interfaces is shown in Figure 7, and the average trap concentration level at the source/drain regions are shown in Figures 8 and 9 respectively.

From Figure 6, we can see that the on-state resistance (R_on_) and off-state drain leakage (refer to right y-axis) current increase with the TLP stress level, while the transconductance (g_m_), threshold voltage (V_th_) and saturation current decrease with the TLP stress level. Under the pulse stress at two-thirds of I_t2_, the on-resistance nearly doubled. The saturation drain current degrades the least among all the device parameters, but the degraded off-state drain leakage current can be thousands of times of that in the degradation-free device. The physical mechanisms of the changes in these device parameters will be explained in the next section.

It is know that the GAA nanowire device has good gate controllability due to the gate stack configuration, and its off-state leakage current is much lower than other device structures [1,5]. However, as we can see here, this advantage is lost when it is subjected to ESD stress, and hence effective ESD protection to the device is critically important to leverage on the strength of the GAA SiNW FET.

The upper bound of interface trap concentration is set as the silicon dangling bond concentration at the Si-SiO_2_ interface (10^12^ cm^-2^ is used in this work [17]). From Figures 7, 8 and 9, we can see that the maximum interface trap concentration increases rapidly at the rising edge of the stress pulse while the average trap concentrations increase relatively slower.

Figure 9 shows that the increase in the average trap concentration at the drain region is similar to the maximum trap concentration increase as shown in Figure 7, indicating that more interface traps are generated at the drain region at the rising stage of the stress pulse, indicating that the mechanism of interface traps generation is due to hot carrier injection instead of bias temperature instability.

After the pulse rising time, the traps increase steadily, and the trap concentration at the drain region interface is much higher than that at the source region interface. These observations will be explained in the next section.

### Degradation analysis

The device performance will be severely degraded due to the reduction of carrier mobility as more scattering occur upon the trap charge formation at the Si-SiO_2_ interface. The degradation is expected to be more severe for the GAA nanowire device as the surface to volume ratio of nanowire body is high.

**Figure 10:**
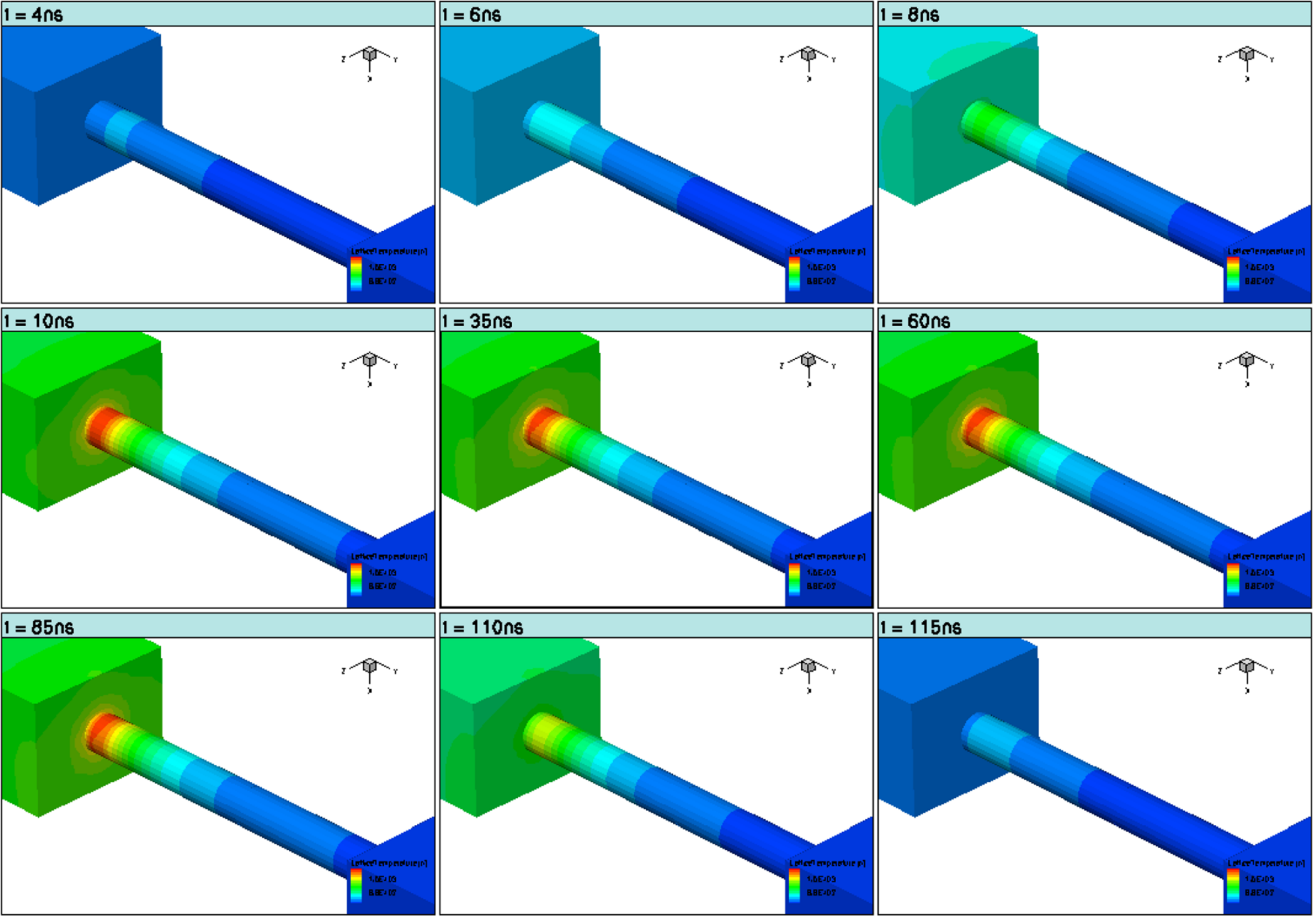
**Time evolution of hotspot position during TLP (The left block is the drain region. The stress time is indicated in each of the frame header).**

Our TLP simulation indicates that the hotspot position is in the drain extension region as shown in Figure 10, and hence the major degradation mechanism in GAA nanowire device should be at the drain junction.

Under the TLP stress condition, the increase in the interface trap charge concentration at Si-SiO_2_ interface enhances the carrier scattering which in turn reduces the carrier mobility and channel current, thus R_on_ rises and g_m_ decreases as observed in Figure 6. The induced interface trap charge in the channel region is also responsible for the threshold voltage shifting [18]. The degraded threshold voltage means less effective gate control, which in turn increase the off-state current dramatically.

With the increase in the electrons trap concentration at the interface, the channel current path moves slightly further away from the interface as reported by Chen et al. [18,19]. This, together with the reduction in the channel current, suppresses the impact ionization and further reduces the generation rate of interface traps due to hot carrier injection (HCI). Therefore, the interface states or degradation reaches certain equilibrium state after some time as observed in Figures 8 and 9 where the average trap concentration maintains at a level after first 50 ns stress. For low level stress, the saturation interface states level is far from the upper limit of interface states, which indicates that there is no more newly created interface traps. If the stress pulse is strong enough, as in the case of 10 mA/μm stress, the maximum interface trap concentration can reach the saturation limit as shown in Figure 7.

**Figure 11:**
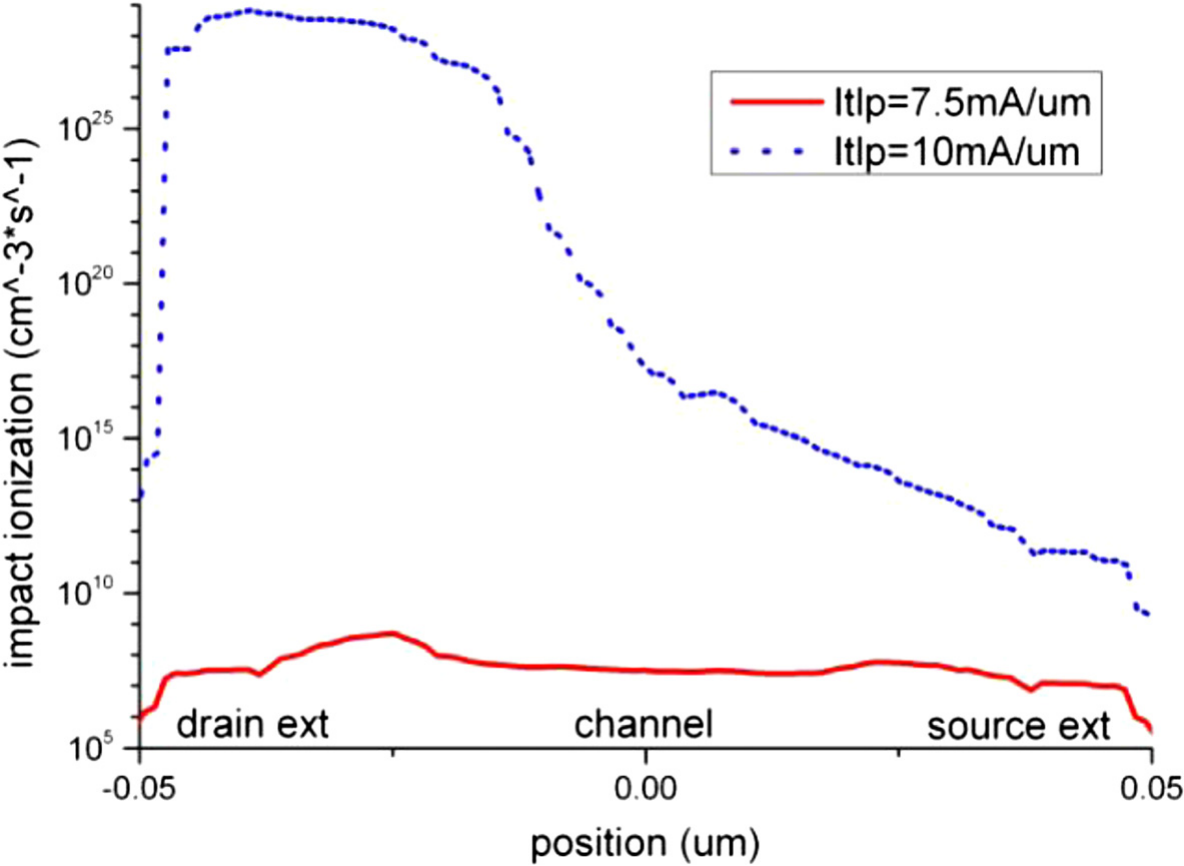
**Impact ionization distributions along the nanowire Si-SiO**_**2**_ **interface.**

**Figure 12:**
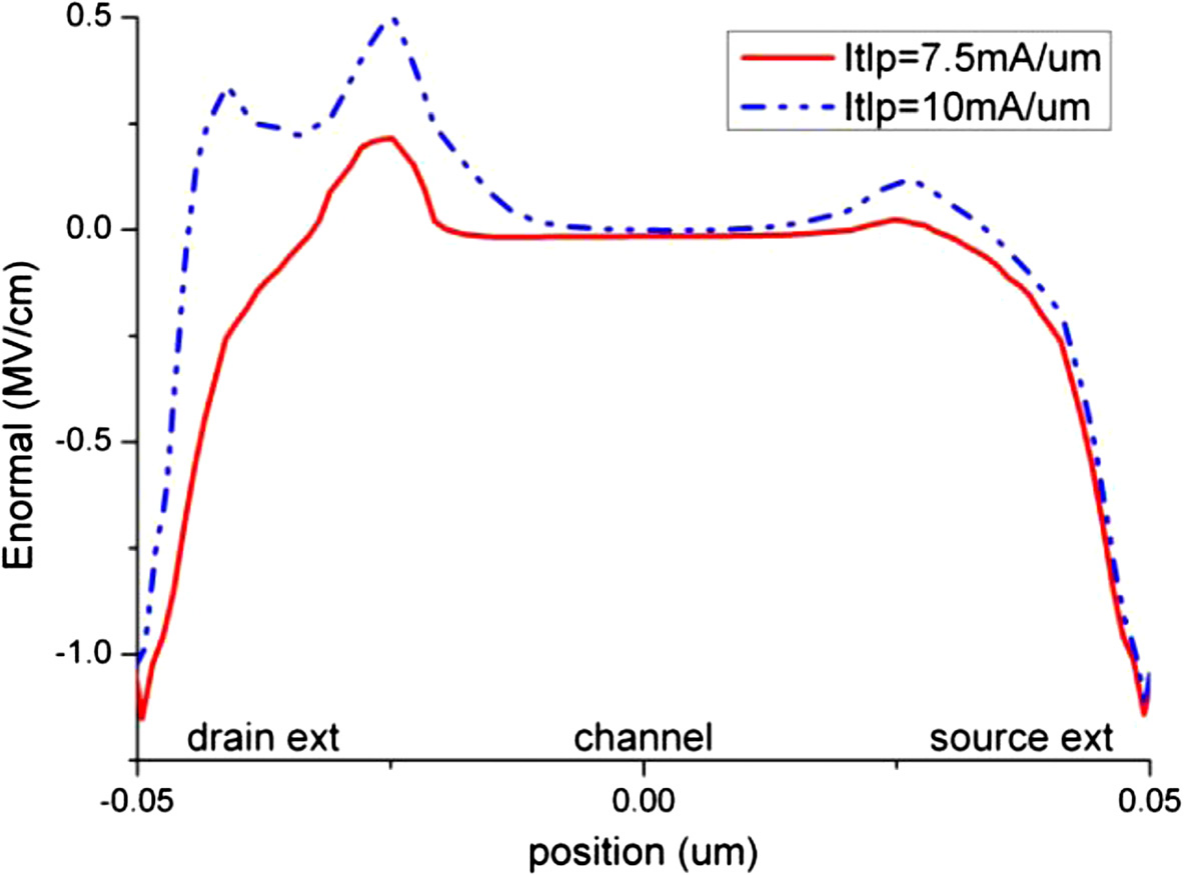
**Distribution of the normal electric field along the nanowire Si-SiO**_**2**_ **interface.**

**Figure 13:**
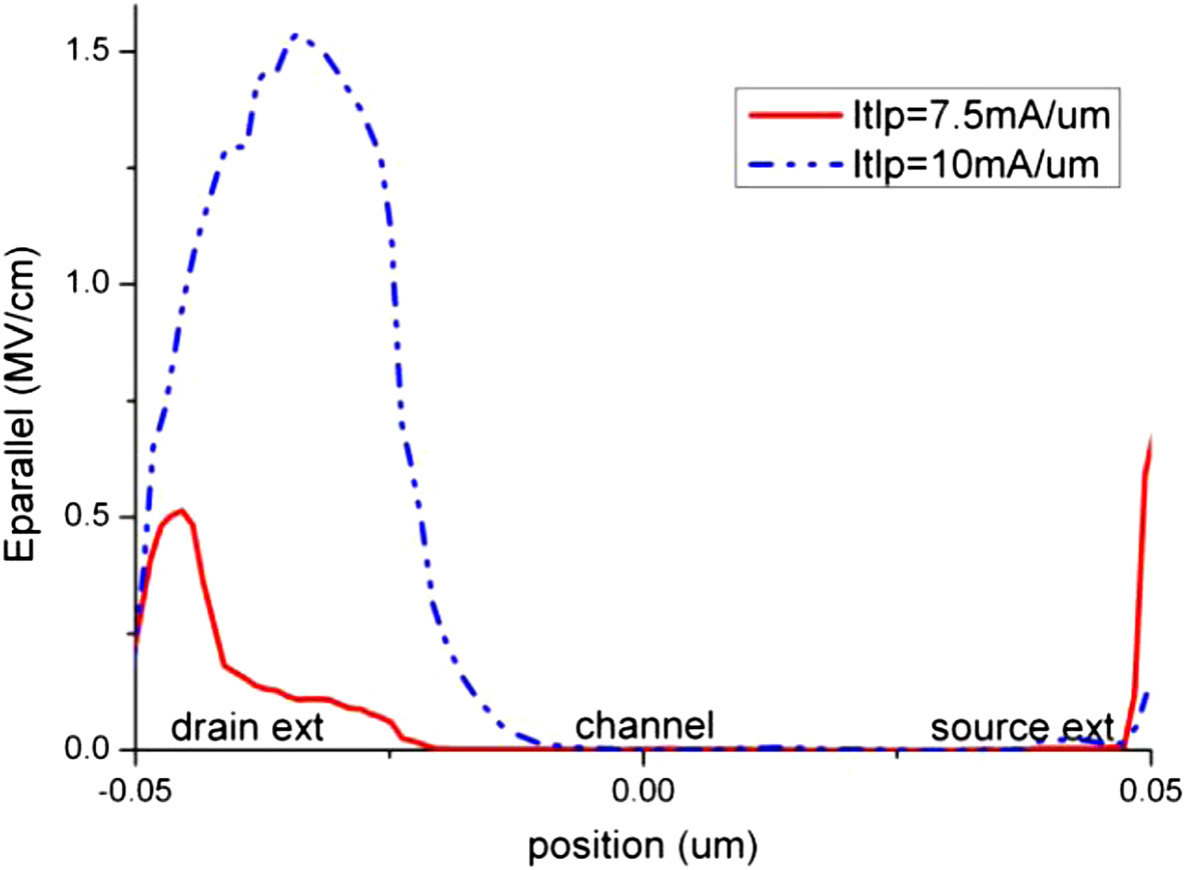
**Distribution of the parallel electric field along the nanowire Si-SiO**_**2**_ **interface.**

The impact ionization generation is nearly absent when the TLP stress level is lower than 7.5 mA/μm, which is half of the I_t2_. Above 7.5 mA/μm stress level, impact ionization generates electron-hole pairs massively at the drain side as shown in Figure 11. The normal electric field shows a peak region at the drain extension, and it also becomes more significant when stress level is above 7.5 mA/μm as depicted in Figure 12. High electron current density and high parallel electric field as shown in Figure 13 also exist at the drain extension, which suggests that more hot electrons injection occur at this position. Therefore, the electron density at the drain side increases rapidly during the pulse rising edge due to the TLP current injection. However the electron current density at the source side does not vary as much as that at the drain side. This explains a sudden rising of interface states at the drain side, while no such effect is observed at the source side.

**Figure 14:**
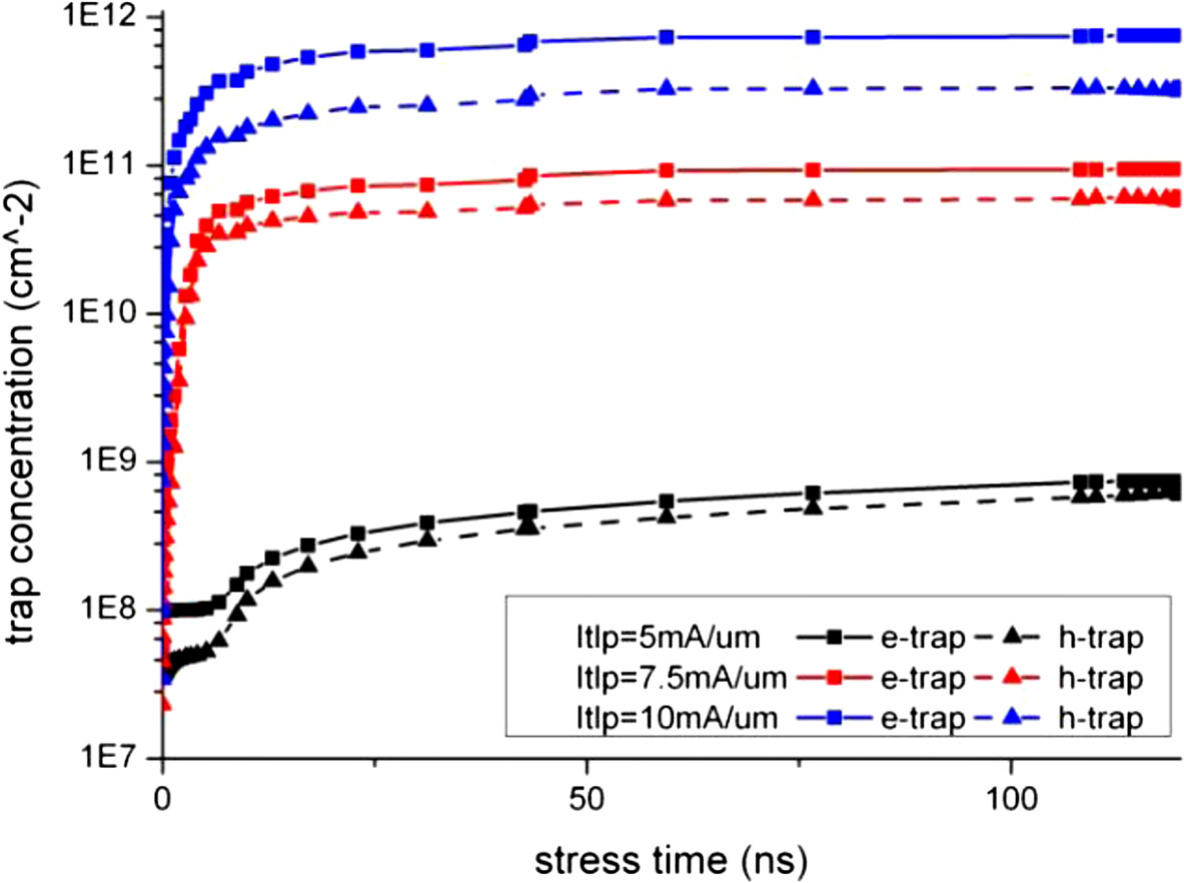
**Electron and hole interface trap concentration under different TLP stress.**

A large negative magnitude of normal electric field also exists at the drain to drain extension and the source to source extension as shown in Figure 12. This negative field suggests a favor for hole injection at these two positions. Trap of holes could induce negative mirror charge near the interface, increase the effective electron concentration. This mechanism results in drain current increase and R_on_ decrease. However such reverse shifting behavior of device electrical parameter is not observed in our simulation since the electron trap is dominating. As the intrinsic p-type doping level of nanowire is much lower than the source/drain doping, there are relatively fewer active holes as compare to electrons, thus the dominant carrier trap and degradation mechanism is hot electron injection related, and the hot hole injection is only a minor competing mechanism in this case. The time progression of electron and hole trap concentration is depicted in Figure 14. With higher stress current level, the electron trap becomes even more effective due to larger positive normal electric field and there are more accumulated electrons, and the impact of the hot hole injection is becoming less as can be seen in Figure 14. Therefore, the R_on_ shifting curve shown in Figure 6 shows an increasing slope with the ESD stress level.

The ESD stress seriously degrades the device performance by generating the charged interface traps, and high level charge traps could reduce the oxide breakdown voltage, and in some cases, the breakdown voltage is so low that local oxide melts due to the local conduction path formed by accumulated oxide traps. This is also reported by Tseng and Hwu [15].

From the above analysis, we can see that ESD TLP stress on the drain of GAA nanowire FET will first trigger the hot carrier injection at Si-SiO_2_ interface which degrades the device performance. If the stress level is high or the stress persists, oxide breakdown will occur, and in some severe cases, oxide melt will be observed. All these degradation mechanisms are indeed observed experimentally in [2].

During our simulations, the gate terminal is kept at floating. Since GAA devices is a fully depletion device, the ESD current mainly discharges through the nanowire channel, and thus their ESD robustness is strongly dependence on the gate voltage. As the gate could also couple the transient voltage from the drain terminal during TLP stress, the ESD damage should be even more significant if the gate is grounded, and this will be investigated in our future work.

## Conclusion

GAA silicon nanowire device is a promising nano structure for next generation semiconductor device, but the deep scaled device features and its gate-all-around configuration make the device to have limited ESD reliability. This work aims to study the device hard breakdown and performance degradation under HBM equivalent ESD stress. Severe ESD stress level could catastrophic melt the device structure due to large amount of local heat generation. Lower stress level also induces significant device performance degradation due to the accumulation of interface traps the drain end of the GAA silicon nanowire FET. This work provides further understanding on the nanowire device degradation mechanism during ESD event.
